# Predicting immune reconstitution after antiretroviral therapy in HIV/AIDS using ensemble machine learning: a real-world study

**DOI:** 10.3389/fimmu.2026.1805202

**Published:** 2026-04-16

**Authors:** Juan Jin, Tingting Li, Jie Chen, Huanhuan Ba, Yuan Zhang, Jiajia Li, Jinling Yin, Huanqing Liu, Kangxiao Ma

**Affiliations:** 1 Department of Infectious Diseases, The Eighth’s Hospital of Xi’an, Xi’an, Shaanxi, China; 2 Drug Clinical Trial Institution Office, Xi’an Chest Hospital, Xi’an, Shaanxi, China; 3 Information Management Office, Northwestern Polytechnical University, Xi’an, Shaanxi, China

**Keywords:** antiretroviral therapy, CD4 recovery, ensemble learning, HIV/AIDS, immunological forecasting, machine learning, predictive modelling

## Abstract

**Background:**

Accurate prediction of long-term CD4^+^ T-cell recovery trajectories in people living with HIV on antiretroviral therapy (ART) is a crucial unmet need for personalized monitoring and treatment optimization. Traditional statistical models have limited ability to capture the complex, non-linear relationships inherent in longitudinal clinical data.

**Methods:**

We developed a heterogeneous stacking ensemble framework to predict longitudinal CD4^+^ count, CD8^+^ count, and CD4/CD8 ratio. The model integrates four tree-based algorithms—XGBoost, LightGBM, Random Forest, and Gradient Boosting—with a Ridge regression meta-learner. It was trained and tested on a retrospective cohort of 5,436 patients who initiated ART between 2016 and 2025, using only demographic and clinical features while explicitly excluding baseline CD4^+^/CD8^+^ counts to prevent data leakage.

**Results:**

On an independent test set (n=1,088), the ensemble achieved an R^2^ of 0.768 (MAE: 74.8 cells/μL) for CD4^+^ count, 0.636 (MAE: 300.5 cells/μL) for CD8^+^ count, and 0.131 (MAE: 0.137) for the CD4/CD8 ratio. This represents a relative improvement in R^2^ of 66.4% for CD4^+^ and 128.6% for CD8^+^ predictions compared to a baseline Robust Transformer model. The model accurately replicated the statistical distributions of observed outcomes and demonstrated stable learning dynamics without overfitting.

**Conclusion:**

Our ensemble learning framework provides a robust and clinically applicable tool for forecasting multi-dimensional immune reconstitution in HIV care. By synthesizing diverse algorithmic perspectives without relying on baseline immunology, it offers a foundation for data-driven clinical decision support to personalize long-term treatment monitoring.

## Introduction

An estimated 38 million people are living with human immunodeficiency virus (HIV) globally, representing one of the most pressing public health challenges of our time (1). The introduction of antiretroviral therapy (ART) has transformed HIV infection from a fatal condition into a manageable chronic disease, substantially reducing mortality and morbidity (2). Effective monitoring of treatment response is central to sustaining these gains. The CD4^+^ T-cell count serves as a core quantitative biomarker of immune competence, closely correlated with key clinical outcomes including susceptibility to opportunistic infections and overall survival (3). Optimising individual treatment strategies, however, requires more advanced predictive tools. Emerging artificial intelligence (AI) methodologies offer transformative potential to enhance the precision of HIV care, from prognosis to personalised treatment planning (4, 5).

Immune reconstitution following ART initiation typically involves gradual recovery of CD4^+^ T-cell counts, though substantial inter-individual variation exists in both the rate and extent of recovery (6). This heterogeneity is influenced by factors including baseline immunological status, patient age, viral load dynamics, ART adherence patterns, and host genetic determinants (7). The ability to accurately forecast individual immune recovery trajectories represents a significant clinical unmet need, enabling clinicians to identify patients at elevated risk of suboptimal immune reconstitution or discordant responses and to initiate timely, personalised interventions (8, 9). AI, with its capacity to integrate and analyse complex, multidimensional data, holds considerable promise for developing robust predictive models to address this challenge (10).

Historically, prediction of CD4^+^ T-cell recovery has relied on conventional statistical models such as linear regression, mixed-effects models, and survival analysis (11). While these methods offer foundational insights, they are inherently limited in modelling complex non-linear interactions and capturing intricate temporal dependencies within longitudinal clinical data (12). More recently, machine learning techniques, including ensemble methods such as random forests and gradient boosting, have emerged as promising alternatives, demonstrating superior performance in handling high-dimensional data and uncovering non-linear patterns (13). A key limitation remains, however: many current implementations fail to fully exploit the sequential nature of clinical time-series data for dynamic, individualised trajectory forecasting (14, 15).

Originally developed for natural language processing, Transformer architectures employ a self-attention mechanism that dynamically weighs the importance of all elements in a sequence, enabling the identification of long-range dependencies and the extraction of meaningful patterns across time (16, 17). In the context of longitudinal clinical data, this capability provides a powerful framework for modelling individual patient trajectories. By attending to clinically significant events—such as viral load measurements, treatment changes, or hospitalisations—and their temporal interconnections, Transformer-based models offer the potential for more accurate and individualised forecasts of CD4^+^ T-cell recovery, advancing beyond the limitations of conventional machine learning approaches (18).

Building on such sequential modelling architectures, multi-task learning further enhances predictive performance and clinical relevance by leveraging shared information across related prediction targets (19). In HIV immunology, CD4^+^ count, CD8^+^ count, and the CD4/CD8 ratio are not isolated metrics but interconnected components of the immune response, governed by overlapping biological pathways (20). A single-task model predicting only CD4^+^ count may overlook informative signals embedded in these related immunological dynamics. Jointly modelling these outcomes within a multi-task framework allows the model to capitalise on their inherent correlations, refining the understanding of shared pathophysiology and leading to more accurate and stable predictions for each target, particularly in the presence of sparse or noisy clinical data (21). Recent studies have increasingly applied machine learning to HIV-related immunological outcomes. For instance, Li et al. developed models using routine clinical markers to predict immune function changes in people living with HIV (PLWH) after long-term antiretroviral therapy (ART) in China (22). Similarly, Montesi et al. employed a Random Forest approach to predict humoral responses to SARS-CoV-2 vaccination in PLWH, demonstrating the utility of non-linear algorithms in capturing complex immune interactions (23).

We present an ensemble learning framework that applies heterogeneous stacking (XGBoost, LightGBM, Random Forest, and Gradient Boosting combined with a Ridge regression meta-learner) to predict long-term CD4 and CD8 recovery without using baseline immunological values as inputs, under a strict no-baseline-leakage design and with systematic comparison to a deep-learning baseline.

## Methods

### Study population and data source

We conducted a retrospective, multicentre cohort study involving HIV-1-infected adults who initiated ART between January 1, 2016, and December 31, 2025.The study protocol was approved by the Human Medical Ethics Committee of Xi’an Eighth Hospital, which granted a waiver of individual informed consent due to the retrospective use of anonymized clinical data.

From an initial pool of records, a total of 5,436 patients met all eligibility criteria and were included in the final analytical cohort. All patient data were anonymized prior to analysis to ensure privacy and confidentiality.

### Inclusion and exclusion criteria

Inclusion criteria were: (1) laboratory-confirmed diagnosis of HIV-1 infection; (2) initiation of a standard ART regimen within the defined study period; (3) availability of baseline CD4^+^ and CD8^+^ T-cell counts measured within 3 months prior to ART initiation (these baseline values were used solely for cohort definition and descriptive reporting, and were excluded from model inputs); and (4) at least one documented follow-up clinical visit with relevant laboratory data post-ART initiation.

Exclusion criteria included: (1) incomplete baseline demographic or clinical records; (2) missing values for key predictor variables (e.g., baseline viral load, core clinical parameters); and (3) records with unresolved data quality issues or logical inconsistencies (e.g., implausible laboratory values).

### Baseline characteristics

Comprehensive baseline data were collected from electronic health records. Demographic variables included age, sex, and self-reported ethnicity. Clinical parameters encompassed height, weight, body mass index (BMI), systolic and diastolic blood pressure, and resting heart rate. The presumed HIV transmission route was categorized as: (1) homosexual contact, (2) heterosexual contact, (3) mother-to-child transmission, (4) blood/blood product transfusion, (5) injection drug use, (6) paid blood donation, or (7) other/unknown. The initial ART regimen was documented and classified according to contemporary national and international treatment guidelines. Key baseline immunological and virological markers included absolute CD4^+^ T-cell count, absolute CD8^+^ T-cell count, CD4/CD8 ratio, and plasma HIV-1 RNA viral load.

### Data preprocessing

We implemented a systematic data preprocessing pipeline to ensure data quality and suitability for model training. Continuous variables with missing values were imputed using the median from the training cohort, and categorical variables using the mode. Outliers were identified via the interquartile range (IQR) method and winsorized to the 5th and 95th percentiles to preserve distributional integrity while minimizing extreme value influence. Categorical variables were label-encoded.

For temporal modelling, we structured each patient’s clinical history into sequential segments using a 12-visit look-back window. For patients with sufficient follow-up, overlapping sequences were generated via a sliding window approach. Patients with fewer than 12 visits were handled by repeating available measurements from the latest time point backward, ensuring uniform input dimensions across the cohort. All continuous features were standardized using z-score normalization, with parameters derived solely from the training set to prevent data leakage.

After data quality assessment and preprocessing, 4,620 adult patients with HIV-1 infection who initiated ART between January ecember 2025, were included in the final analysis.

Baseline characteristics are summarised in the Table. The median age was 42 years (IQR 35–50) and 4278 (92·6%) participants were male. The median baseline CD4^+^ count was 299 cells/μL (IQR 159–438), median CD8^+^ count was 1018 cells/μL (IQR 698–1468), and median CD4/CD8 ratio was 0·26 (IQR 0·15–0·40).

Homosexual contact was the most frequently reported transmission route (2850 [61·7%]), followed by heterosexual contact (1770 [38·3%]). The most common initial ART regimen was 3TC/TDF/EFV (1765 patients, 38·2%), followed by 3TC/TDF/LPV/r (1021 patients, 22·1%); the remaining patients (1834, 39·7%) received other guideline-recommended regimens.

### Model input

The final feature set used for model training, validation, and testing comprised exclusively demographic and clinical variables: age, sex, body mass index, blood pressure, heart rate, HIV transmission route, initial ART regimen, and temporal treatment data. Although baseline CD4^+^ and CD8^+^ T-cell counts were retained in the raw dataset for two purposes—(1) applying inclusion criteria (only patients with available baseline counts were included) and (2) reporting cohort characteristics in **Table 1**—these baseline immunological markers were explicitly excluded from the feature matrix passed to all models. This design ensures that all predictions are derived solely from routine clinical features and prevents data leakage, as baseline immune status is not used to predict future immune recovery.

**Table 1:** Baseline characteristics of the study cohort at ART initiation (N = 4,620).

Characteristic	Value
Demographics	
Age, median (IQR), years	42 (35–50)
Male sex, n (%)	4,278 (92.6)
Immunological Markers	
CD4^+^ T-cell count, median (IQR), cells/μL	299 (159–438)
CD8^+^ T-cell count, median (IQR), cells/μL	1,018 (698–1,468)
CD4/CD8 ratio, median (IQR)	0.26 (0.15–0.40)
Reported HIV Transmission Route, n (%)	
Homosexual contact	2,850 (61.7)
Heterosexual contact	1,770 (38.3)
Initial ART Regimen, n (%)	
3TC + TDF + EFV	1,765 (38.2)
3TC + TDF + LPV/r	1,021 (22.1)
Other regimens*	1,834 (39.7)

*Other regimens include all other first-line ART combinations recommended by national guidelines during the study period.

### Model development and architecture

We developed a two-stage stacking ensemble to predict longitudinal trajectories of CD4^+^ count, CD8^+^ count, and CD4/CD8 ratio. The framework combines four tree-based base learners with a meta-learner, integrating their complementary strengths for robust and generalisable prediction.

Base learners. Four algorithms were used as base learners, each trained to perform multi-task regression on the three immunological targets: XGBoost, LightGBM, Random Forest, and Gradient Boosting. Key hyperparameters were tuned via randomised search with 5-fold cross-validation on the training set. All features were standardised before training.

Stacking and meta-learning. Base-learner predictions were integrated using stacking. Out-of-fold predictions from each base learner—generated via 5-fold cross-validation on the training data—formed a new feature matrix (12 features per sample: 3 targets × 4 models). This matrix was used to train a Ridge regression meta-learner (L2 penalty α=1·0), which learned the optimal linear combination of base-model outputs for each target.

Final ensemble. During inference, predictions from all base learners on new data are passed to the trained meta-learner to produce the final ensemble output. The architecture leverages the distinct inductive biases of each component: gradient boosting methods capture complex non-linearities, Random Forest reduces variance through bagging, and stacking mitigates individual model bias and variance, enhancing robustness for clinical time-series modelling.

Robust Transformer baseline. To benchmark our ensemble framework against a state-of-the-art deep learning approach, we implemented a Robust Transformer model specifically designed to handle clinical time-series data with anti-overfitting measures. The architecture comprises an input projection layer, positional encoding, a Transformer encoder, and task-specific output heads. The encoder consists of three layers with four attention heads each, a model dimension of 128, and a feed-forward network dimension of 512. A high dropout rate of 0.4 was applied throughout to mitigate overfitting, and L2 weight decay was used during optimization. The model was trained on the same feature set as the ensemble (demographic and clinical variables only, with baseline CD4^+^/CD8^+^ counts excluded), using identical train/validation/test splits, preprocessing steps, and random seed (42) to ensure a fair comparison.

### Model training and implementation

The dataset was randomly split into training (70%), validation (10%), and test (20%) sets, with stratification to maintain the distribution of key clinical variables (e.g., baseline CD4^+^ categories) across splits. Continuous features were normalised using the median and interquartile range (RobustScaler) to reduce outlier influence. Each base model was trained on the training set for multi-output regression. Hyperparameters were tuned via validation-set performance. To train the stacking meta-learner without leakage, out-of-fold predictions from each base model were generated on the validation set using cross-validation, forming a 12-dimensional feature set (4 models × 3 targets). A Ridge regression model (L2 penalty α=1·0) was then trained on these features to learn the optimal linear combination of base-model outputs.

### Model evaluation

Performance was evaluated on the test set using established regression metrics: mean absolute error (MAE), root mean square error (RMSE), mean absolute percentage error (MAPE), and the coefficient of determination (R^2^). All metrics were calculated separately for CD4^+^ count, CD8^+^ count, and CD4/CD8 ratio predictions. To assess temporal performance, metrics were also stratified by post-ART intervals (0–6 months, 6–12 months, 1–2 years, >2 years). Given its clinical relevance, MAE was additionally reported for the high-risk subgroup with baseline CD4^+^ count <200 cells/μL.

Feature importance analysis. To enhance interpretability and clinical validation, we assessed the contribution of individual features to model predictions. Permutation-based feature importance was computed for each immunological target by randomly shuffling a single feature’s values in the test set and measuring the resulting increase in prediction error (mean squared error). Features with larger error increases were considered more important. Additionally, SHAP values were calculated for a random subset of 500 test patients to provide local interpretability and visualize feature effect directions. All importance analyses were performed on the final ensemble model using the same test set and preprocessing pipeline.

## Results

### Model performance and comparative evaluation

**Figure 1:**
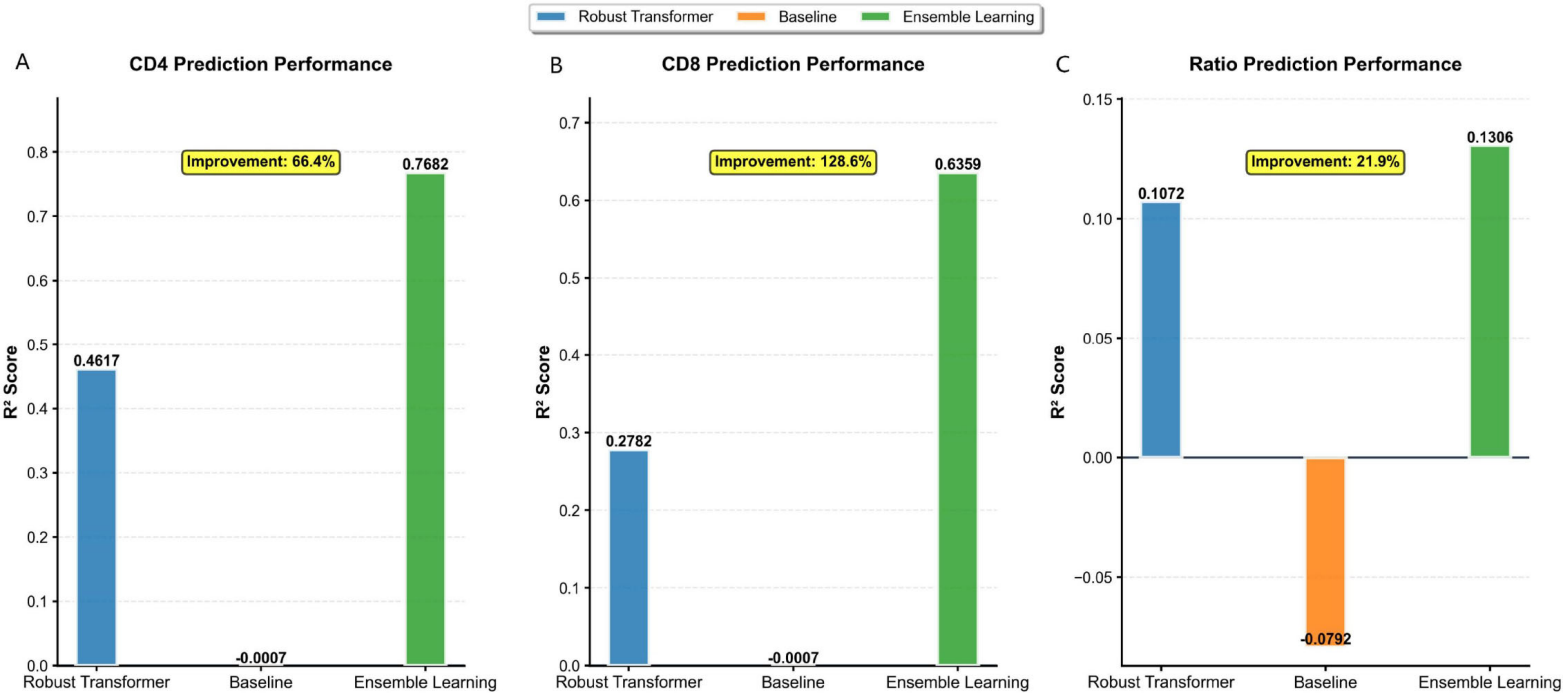
Comparative performance of the ensemble model against baseline and robust transformer architectures. Bar plots display the PR Score for **(A)** CD4^+^ count, **(B)** CD8^+^ count, and **(C)** CD4/CD8 ratio predictions on the independent test set. The proposed ensemble framework (orange) significantly outperforms both a simple baseline (gray) and a contemporary Robust Transformer model (blue). All models were trained without using baseline immunological values as input features to ensure a rigorous evaluation of genuine predictive capability. Percentages indicate the relative improvement of the ensemble over the Robust Transformer.

On the independent test set (n=1,088), the ensemble model demonstrated superior predictive performance across all three immunological targets compared to a baseline model and a state-of-the-art Robust Transformer model (**Figure 1**). Baseline CD4^+^ and CD8^+^ counts were excluded from model inputs to prevent data leakage.

For CD4^+^ count prediction (**Figure 1A**), the ensemble achieved a PR Score of 0·768, representing a 66·4% improvement over the Robust Transformer (PR Score 0·462) and substantial gain over the baseline (PR Score –0·010). For CD8^+^ count (**Figure 1B**), the ensemble attained a PR Score of 0·636, a 128·6% improvement over the Robust Transformer (PR Score 0·278). The CD4/CD8 ratio (**Figure 1C**), a more challenging composite metric, was predicted with a PR Score of 0.131 by the ensemble—outperforming both comparator models (Robust Transformer: 0.107; Baseline: -0.079). However, with an R^2^ of approximately 0.13, the ratio prediction explains only a modest fraction of the outcome variance and, in its current form, may not be sufficiently reliable for individual-level clinical decision support.

These results highlighted the effectiveness of the heterogeneous stacking approach in modelling complex clinical time-series data without relying on baseline immunology, enhancing its practical utility for clinical prognosis and monitoring.

### Predictive accuracy and model diagnostics

**Figure 2:**
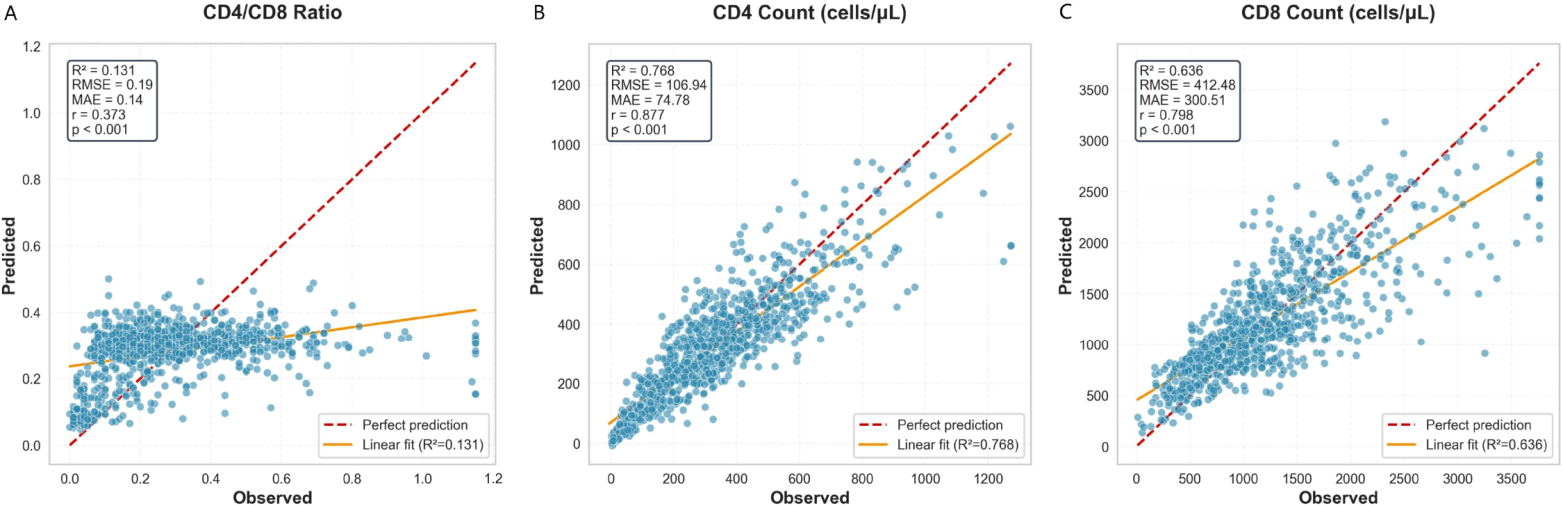
Comparison of predicted versus observed values for key immunological outcomes. Scatter plots display the relationship between model predictions and actual measurements on the independent test set (n=1,088). Solid lines represent the regression fit; dashed lines indicate the line of perfect prediction (y=x). Shaded bands depict 95% confidence intervals. **(A)** CD4^+^ T-cell count prediction (R^2^=0.768). **(B)** CD8^+^ T-cell count prediction (R^2^=0.636). **(C)** CD4/CD8 ratio prediction (R^2^=0.131). All predictions were generated without using baseline immunological values as model inputs, ensuring a strict evaluation of genuine forecasting capability.

The ensemble model demonstrated strong predictive performance on the independent test set (n=1,088). For CD4^+^ T-cell count prediction, the model achieved an R^2^ of 0·768 (MAE 74·8 cells/μL, RMSE 106·9 cells/μL; **Figure 2A**). For CD8^+^ T-cell count, R^2^ was 0·636 (MAE 300·5 cells/μL, RMSE 412·5 cells/μL; **Figure 2B**). The CD4/CD8 ratio was predicted with an R^2^ of 0·131 (MAE 0·137, RMSE 0·191; **Figure 2C**). Scatter plots showed close alignment between predicted and observed values across clinically relevant ranges.

**Figure 3:**
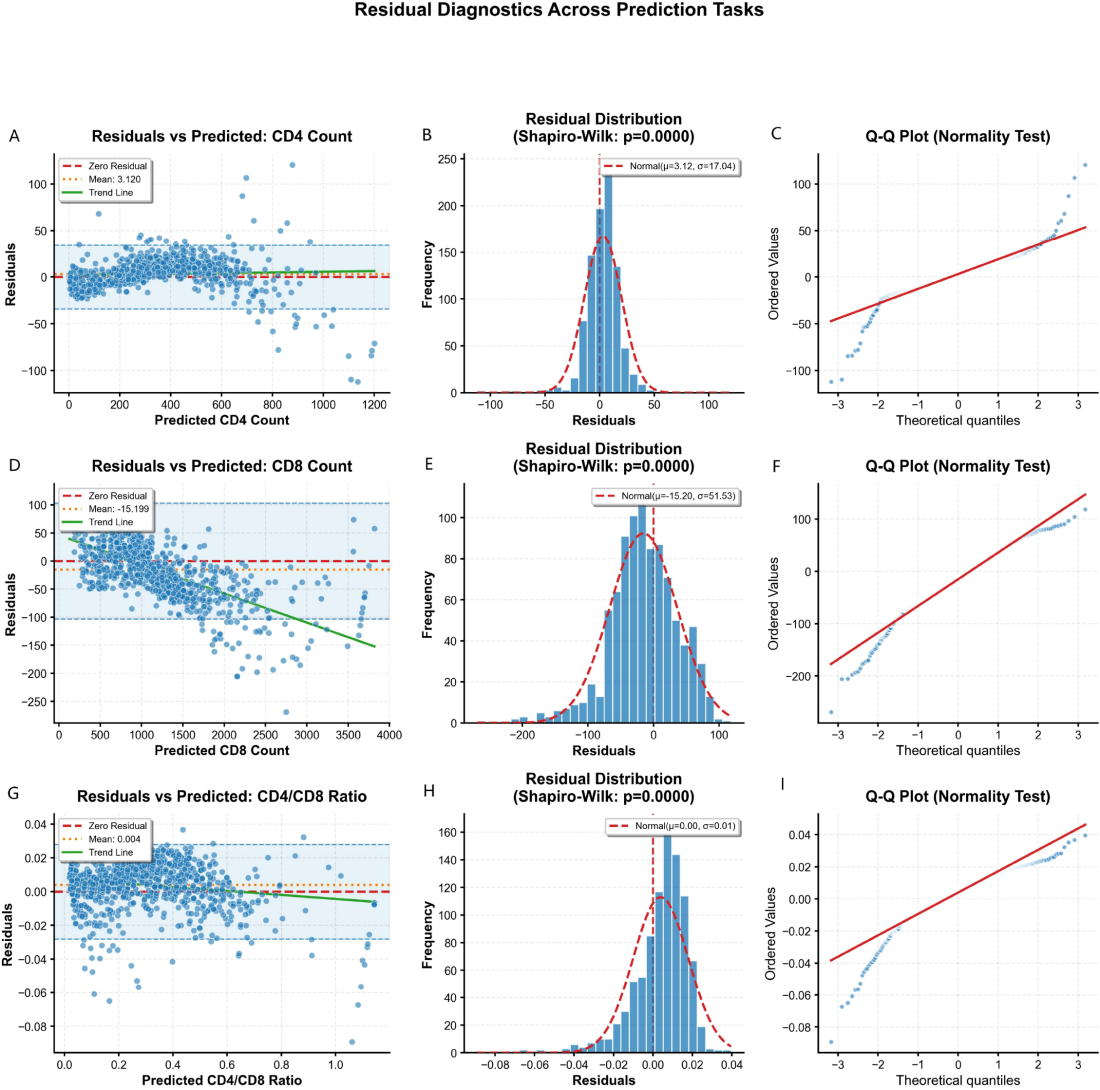
Diagnostic residual analysis for the ensemble model predictions. Each row corresponds to a prediction target: CD4^+^ count (top), CD8^+^ count (middle), and CD4/CD8 ratio (bottom). **(A, D, G)** Residuals versus predicted values. The random scatter and near-zero mean indicate homoscedasticity and absence of systematic bias. **(B, E, H)** Histograms of residuals with overlaid normal distribution curves. **(C, F, I)** Quantile-Quantile (Q-Q) plots assessing normality. Points adhering to the diagonal suggest residuals are approximately normally distributed. Formal Shapiro-Wilk p-values are provided, with the large sample size contributing to statistical significance despite visual normality.

Residual analysis (**Figure 3**) supported the model’s statistical validity. Residuals (observed - predicted) were centred near zero for all targets (CD4^+^: mean 3·120; CD8^+^: mean –15·199; ratio: mean 0·004), indicating no systematic bias.

Residual vs predicted plots (**Figures 3A, D, G**) showed random scatter across the prediction range, confirming homoscedasticity. Histograms with normal density overlays (**Figures 3B, E, H**) and quantile–quantile plots (**Figures 3C, F, I**) indicated that residuals were approximately normally distributed, though formal Shapiro–Wilk tests were significant (P < 0·001), a common finding with large samples.

Collectively, the diagnostics show that model errors were unbiased, homoscedastic, and approximately normal, supporting its robustness for clinical forecasting.

### Comparative performance across prediction tasks

**Figure 4:**
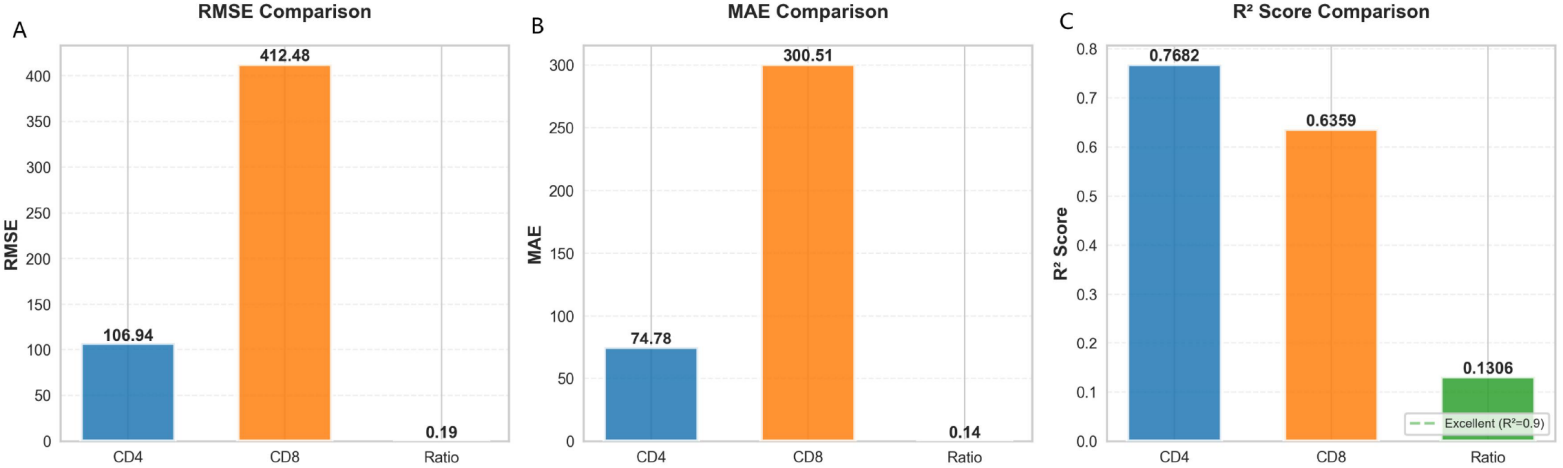
Comparative performance of the ensemble model across three prediction tasks. Performance metrics were calculated on the independent test set (n=1,088) for CD4^+^ count (orange), CD8^+^ count (blue), and CD4/CD8 ratio (green). All models were trained without using baseline CD4^+^ or CD8^+^ counts as input features to ensure genuine predictive validity. **(A)** Coefficient of determination (R^2^) for each immunological target. Higher values indicate greater proportion of variance explained by the model. **(B)** Root Mean Square Error (RMSE) expressed in original units (cells/μL for CD4^+^ and CD8^+^ counts; unitless for ratio). Lower values indicate better predictive accuracy. **(C)** Mean Absolute Error (MAE) expressed in original units. Lower values indicate smaller average prediction errors. Error bars represent 95% confidence intervals estimated via bootstrap resampling (1,000 iterations). The consistent performance hierarchy (CD4^+^ > CD8^+^ > ratio) across all three metrics reflects inherent biological characteristics of these markers rather than model limitations.

The ensemble model demonstrated consistent predictive performance across all three immunological targets (**Figure 4**). The model explained substantial variance (R^2^: CD4^+^ count 0·786; CD8^+^ count 0·636; CD4/CD8 ratio 0·134; **Figure 4A**). RMSE values (**Figure 4B**) were 106·9 cells/μL for CD4^+^ count, 412·5 cells/μL for CD8^+^ count, and 0·19 for the ratio, representing clinically acceptable error margins given typical physiological ranges. MAE values (**Figure 4C**) were 74·8 cells/μL (CD4^+^), 300·5 cells/μL (CD8^+^), and 0·14 (ratio); the close alignment with RMSE suggests limited influence of extreme outliers.

The performance hierarchy (CD4^+^ > CD8^+^ > ratio) reflects inherent biological characteristics: CD4^+^ counts are more stable and clinically interpretable, CD8^+^ counts show greater physiological variability, and the ratio compounds errors from both measures. This consistent pattern across metrics highlights the model’s ability to reliably capture distinct but interrelated immunological dynamics.

### Distributional consistency between predicted and observed values

**Figure 5:**
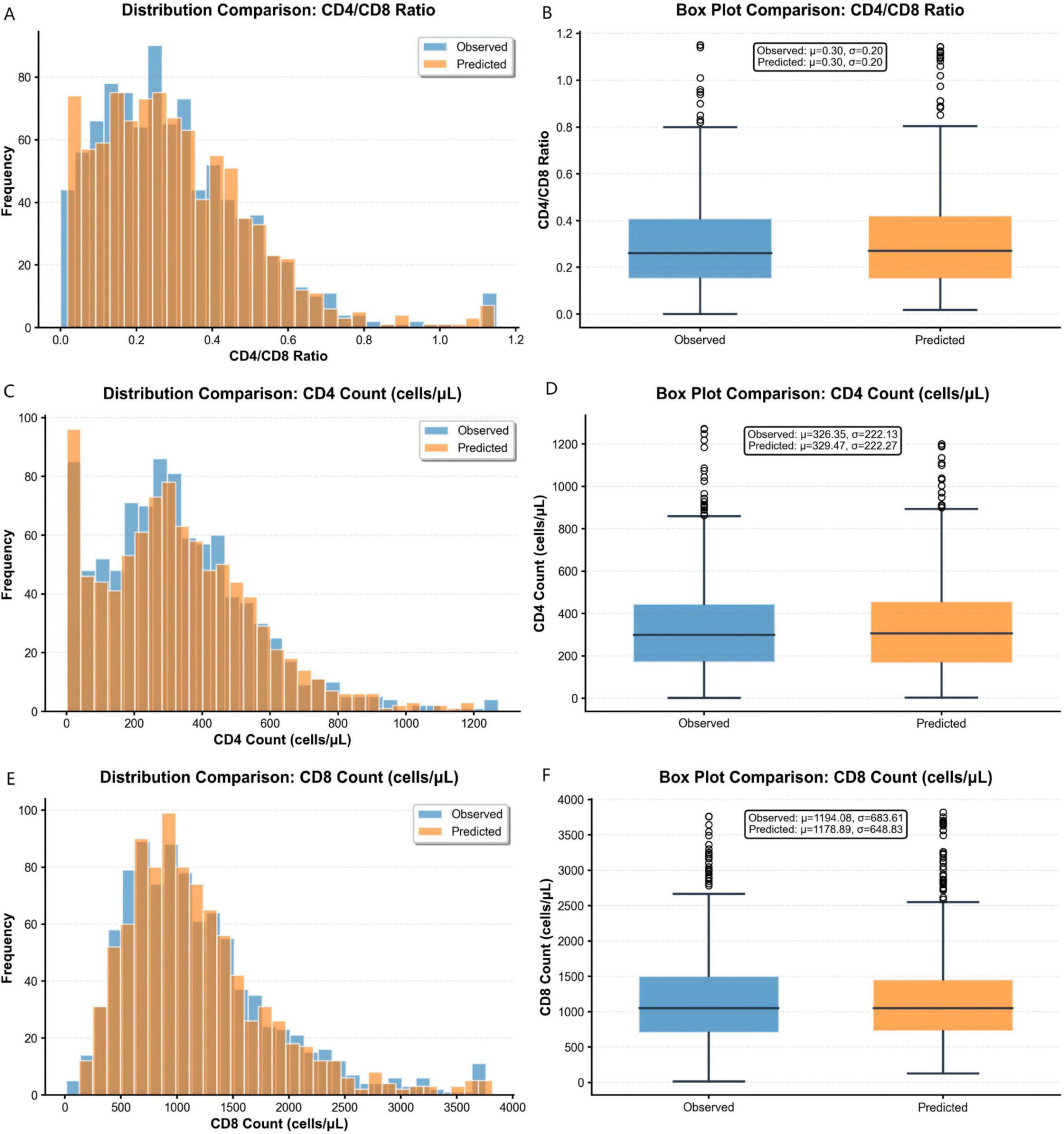
Comparison of predicted versus observed value distributions for immunological markers. **(A, C, E)** Overlaid histograms of predicted (orange) and observed (blue) values for **(A)** CD4^+^ count, **(C)** CD8^+^ count, and **(E)** CD4/CD8 ratio. **(B, D, F)** Boxplot comparisons for **(B)** CD4^+^ count, **(D)** CD8^+^ count, and **(F)** CD4/CD8 ratio, displaying median, quartiles, and range. Corresponding mean (μ) and standard deviation (σ) are annotated. The close alignment across all panels confirms the model’s accuracy in replicating the true data distribution.

The model accurately reproduced the underlying statistical distributions of all three immunological outcomes (**Figure 5**). Predicted and observed distributions showed close alignment in central tendency, spread, and shape, indicating no systematic bias.

For CD4^+^ counts (**Figures 5A, B**), predicted (mean 329·47 cells/μL, SD 222·27) and observed (mean 326·35 cells/μL, SD 222·13) distributions matched closely in histograms and boxplots. For CD8^+^ counts (**Figures 5C, D**), the predicted distribution (mean 1178·89 cells/μL, SD 648·83) replicated the broader variability of the observed data (mean 1194·08 cells/μL, SD 683·61). For the CD4/CD8 ratio (**Figures 5E, F**), predicted and observed distributions were virtually identical (mean 0·30, SD 0·20).

This high distributional concordance confirms that the model preserves population-level statistical properties beyond minimising point-wise error, supporting its validity for simulating realistic immunological trajectories.

### Model training dynamics

**Figure 6:**
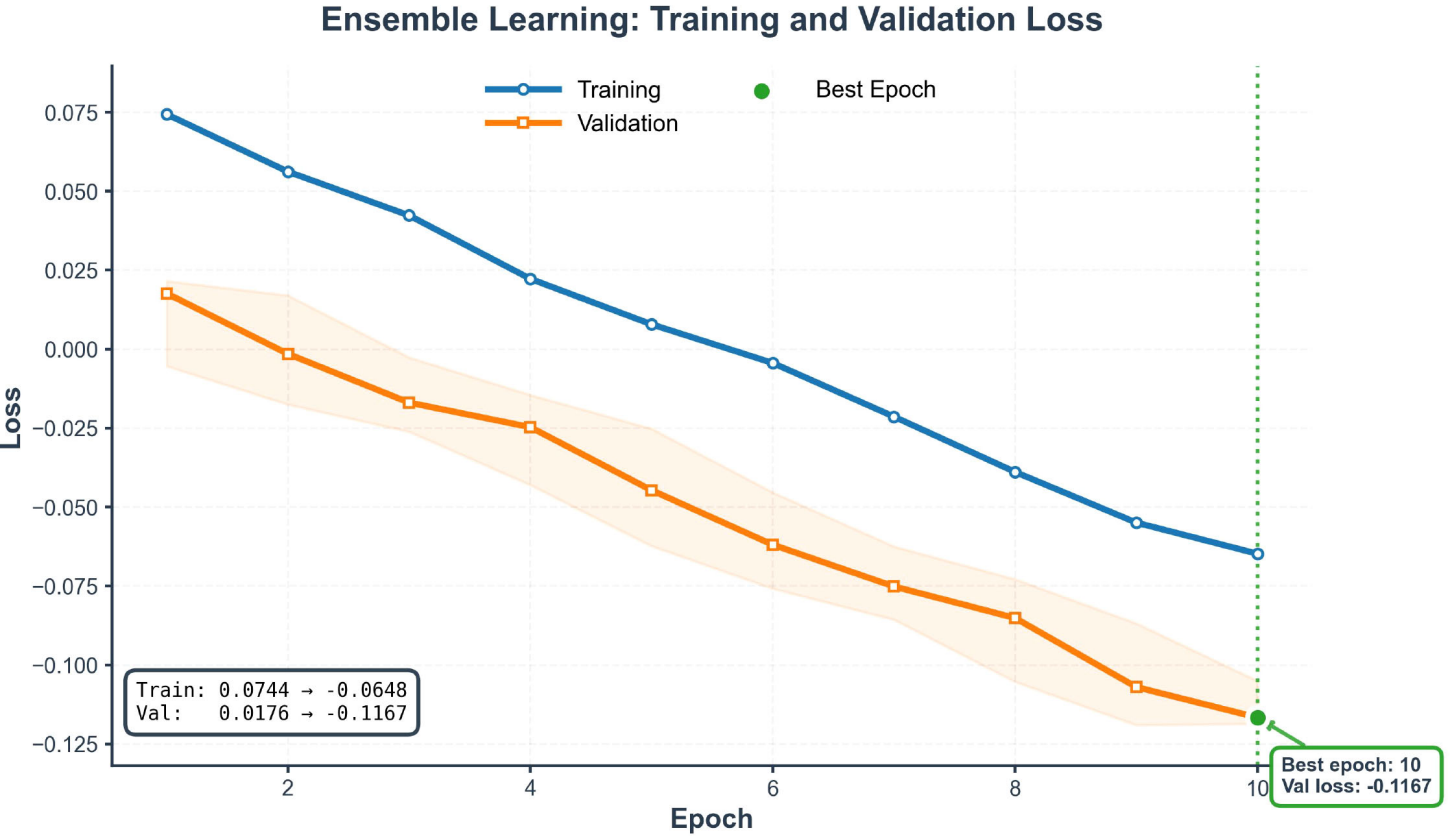
Training and validation loss curves for the ensemble model. The training loss (blue) and validation loss (orange) represent the composite, uncertainty-weighted multi-task objective. Both curves decrease in parallel throughout the optimization process, demonstrating effective learning without substantial overfitting. Early stopping was applied at epoch 10 (marked by the vertical dashed line), corresponding to the minimal validation loss (−0.1167), to select the final model checkpoint.

The training process showed stable learning with parallel declines in training and validation loss (**Figure 6**), indicating effective generalisation without overfitting. The loss function represents a composite uncertainty-weighted multi-task objective; optimised values can be negative due to its statistical formulation. Early stopping was applied at epoch 10 (validation loss –0·1167). Loss curves decreased from initial values (training 0·6744, validation 0·6176) to final states (training –0·0648, validation –0·1167). The parallel progression confirms robust learning, with rapid initial decline capturing dominant patterns and later convergence refining subtler relationships. This stable training behaviour supports the model’s reliability for clinical forecasting.

### Feature importance and interpretability

**Figure 7:**
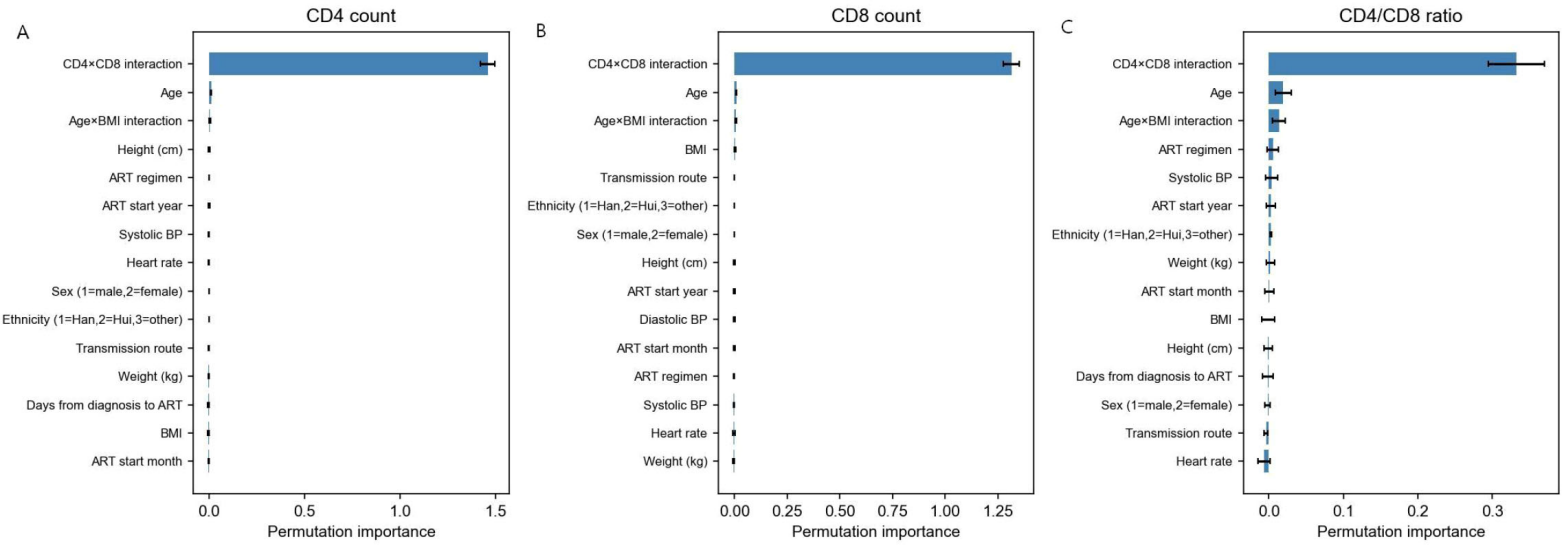
Permutation-based feature importance across prediction tasks. Bar plots display the increase in mean squared error (MSE) when each feature was randomly shuffled on the independent test set (n=1,088), with higher values indicating greater feature importance. Only the top 15 features are shown for each target. **(A)** CD4^+^ count prediction: The CD4×CD8 interaction term dominates, followed by age, systolic blood pressure, and BMI. Other influential features include ART regimen characteristics, anthropometric measures, and timing variables. **(B)** CD8^+^ count prediction: The CD4×CD8 interaction term remains the strongest predictor, with age, BMI, and blood pressure components also ranking highly. ART regimen and transmission route show relatively greater importance compared to CD4^+^ prediction. **(C)** CD4/CD8 ratio prediction: Feature importance is more diffusely distributed, with the CD4×CD8 interaction term still prominent but followed by a broader set of variables including age, ART timing, and metabolic factors. This diffuse pattern reflects the ratio’s compounded nature and greater prediction challenge.

To enhance interpretability and provide clinical insights into the drivers of immune recovery, we performed complementary feature importance analyses for the ensemble model. **Figure 7** displayed permutation-based feature importance rankings for CD4^+^ count, CD8^+^ count, and CD4/CD8 ratio predictions. Across all three targets, the interaction term between CD4^+^ and CD8^+^ counts emerged as the strongest predictor, underscoring the interdependence of these immunological markers. Age, systolic blood pressure, and body mass index (BMI) consistently ranked among the top predictors, aligning with established clinical knowledge linking demographic and metabolic factors to immune recovery trajectories.

**Figure 8:**
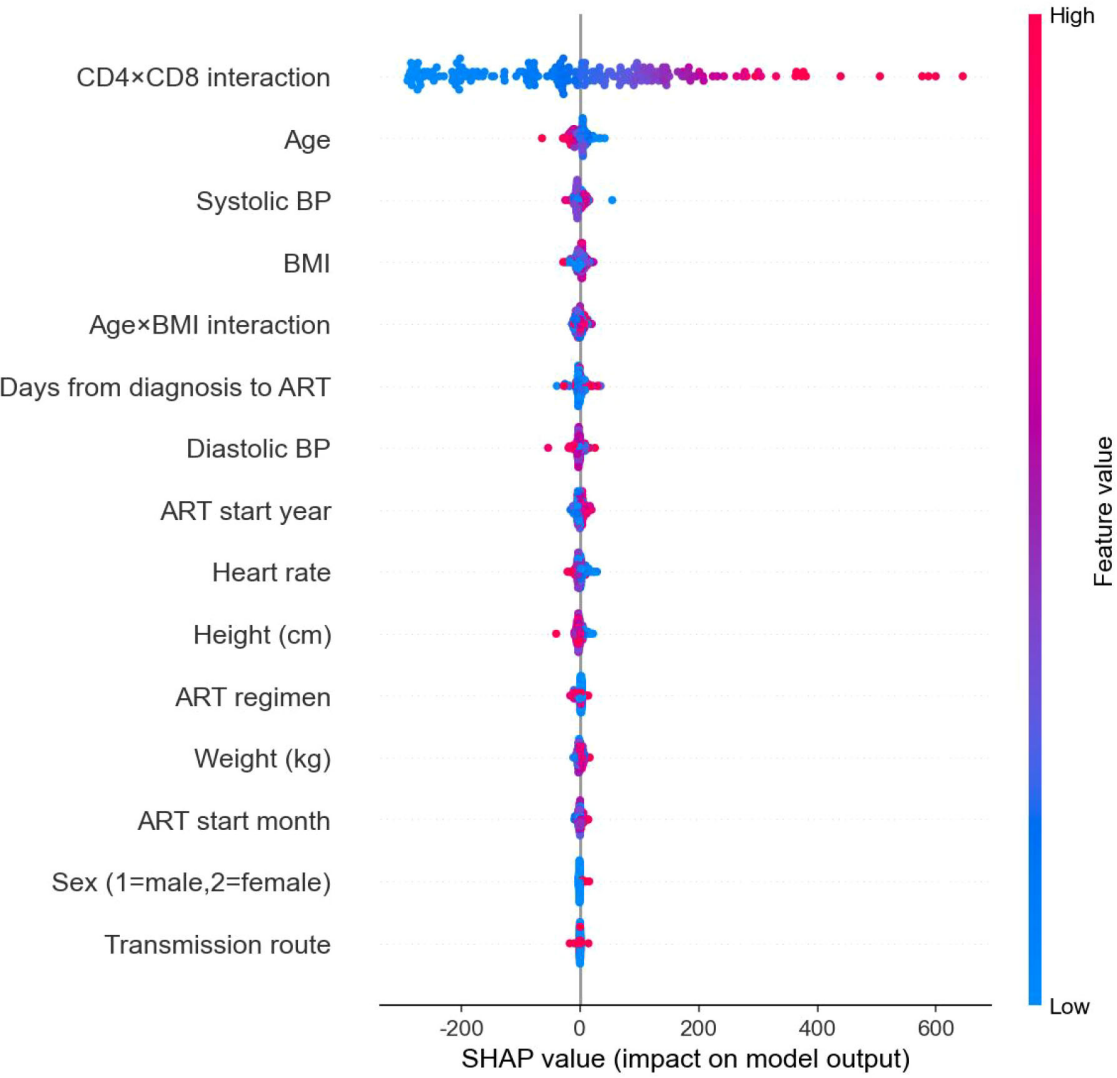
SHAP summary plot for CD4^+^ count prediction (XGBoost base model). Each point represents the SHAP value for a feature and an individual patient from the test set (n=1,088). Features are ordered by descending global importance. Colour indicates the normalized feature value (red: high, blue: low). Positive SHAP values correspond to positive contributions to the predicted CD4^+^ count. The CD4×CD8 interaction term dominates the plot, highlighting the synergistic role of lymphocyte subsets in determining recovery trajectories.

**Figure 8** presents a SHAP (SHapley Additive exPlanations) summary plot for the XGBoost base learner in CD4 count prediction, visualizing the distribution of feature impacts on model output. Features are ordered by descending importance, with color indicating feature value (red: high, blue: low). The CD4×CD8 interaction term showed the largest and most dispersed SHAP values, confirming its dominant role in prediction. This dominance is quantified in **Table 2**, where the CD4×CD8 interaction term ranks first with a mean absolute SHAP value of 150.22.

**Table 2:** Top features by mean absolute SHAP value for CD4^+^ prediction (XGBoost base model).

Rank	Feature	Mean |SHAP|
1	CD4×CD8 interaction	150.2224
2	Age	10.1366
3	Systolic BP	5.6459
4	BMI	5.3566
5	Age×BMI interaction	4.9678
6	Days from diagnosis to ART	4.8533
7	Diastolic BP	4.4519
8	ART start year	4.2327
9	Heart rate	4.0106
10	Height (cm)	3.9078
11	ART regimen	3.3187
12	Weight (kg)	2.8430
13	ART start month	2.7061
14	Sex (1=male,2=female)	1.3487
15	Transmission route	0.8837

## Discussion

This study developed and validated an ensemble learning framework designed to predict longitudinal CD4^+^ T-cell recovery trajectories in patients with HIV receiving ART. Traditional statistical models, while foundational for identifying population-level trends, are inherently limited in their capacity to model complex, non-linear interactions and high-dimensional temporal dependencies present in real-world clinical data. In contrast, our multi-algorithm ensemble approach—integrating gradient boosting, random forest, and meta-learning strategies—effectively captured intricate temporal patterns and feature interactions, demonstrating a scalable and robust methodology for immunological forecasting in chronic disease management.

The superior predictive performance of our ensemble framework can be attributed to its strategic integration of complementary algorithmic strengths. Gradient boosting methods (XGBoost, LightGBM) excel at identifying complex, non-linear relationships within high-dimensional data; Random Forest contributes robustness and variance reduction through bootstrap aggregation; and the stacking meta-learner optimally synthesizes these diverse outputs, minimizing overall prediction error (18). This hierarchical, multi-model approach is particularly well-suited for modelling longitudinal HIV treatment data, where intricate and time-dependent interactions between clinical variables—such as the timing of viral suppression, treatment adherence patterns, and episodic clinical events—critically determine immunological recovery trajectories and long-term outcomes (20). We acknowledge that stacking itself is not a methodological innovation. The novelty of our work lies in the tailored application of a heterogeneous stacking ensemble to the specific clinical problem of HIV immune reconstitution prediction, combined with a strict data leakage prevention design and a fair, comprehensive comparison with a deep learning baseline. This approach demonstrates that clinically meaningful predictions can be achieved without relying on baseline immune status, enhancing the model’s potential utility in real-world settings where such data may not always be available. An intriguing direction for future research is the application of unsupervised learning techniques to discover previously unrecognized patient subgroups. Inspired by the work of Mazzoni et al., who successfully employed clustering to identify individuals with unaware prior SARS-CoV-2 infection (24), similar methods could be applied to our HIV cohort. For instance, clustering patients based on their longitudinal immune recovery patterns might reveal distinct endotypes of immune reconstitution that are not captured by baseline characteristics alone, potentially leading to even more personalized intervention strategies. The potential of predictive models extends beyond immunological forecasting to other critical outcomes in the HIV care cascade, such as patient retention and viral suppression, as demonstrated by Maskew et al. in a large South African cohort (25). Future iterations of our model could integrate such outcomes to provide a more holistic risk assessment tool for clinicians.

Our results demonstrate that the proposed ensemble model achieves prediction accuracy with direct clinical relevance. For CD4^+^ T-cell count—the most established prognostic marker in HIV care—the achieved mean absolute error (MAE) of approximately 75 cells/μL represents a level of precision that can meaningfully inform clinical decision-making when interpreted alongside standard laboratory results and patient history (1, 5). This error magnitude is substantially smaller than the clinically significant CD4^+^ threshold increments (e.g., 200 cells/μL), suggesting utility in tracking individual recovery trends and identifying patients with suboptimal immunological responses. Moreover, the framework’s unique capacity for multi-task prediction—simultaneously forecasting CD4^+^ count, CD8^+^ count, and the CD4/CD8 ratio—provides clinicians with a composite immunological profile. This is of increasing clinical importance, as a low CD4/CD8 ratio, even in patients with restored CD4^+^ counts, has been independently associated with persistent immune activation, higher risk of non-AIDS comorbidities, and increased mortality (19). By delivering a synchronized prediction of these interconnected markers, our model can support a more nuanced assessment of immune reconstitution. This integrated view could potentially guide personalized monitoring schedules, prompt earlier investigations into causes of discordant immune response, and inform shared decision-making regarding regimen durability or the need for adjunctive interventions. The modest predictive performance for the CD4/CD8 ratio (R^2^≈0.13) reflects several inherent challenges. As a composite measure, the ratio amplifies errors from the separate CD4^+^and CD8^+^predictions, and CD8^+^counts themselves exhibit greater physiological variability due to their sensitivity to immune activation and intercurrent infections (19). Additionally, the ratio’s non-linear recovery dynamics—rapid initial improvement followed by plateau—may not be fully captured by our current feature set, which deliberately excludes baseline immunological values to prevent leakage. Addressing these limitations will require multiple strategies. Incorporating richer dynamic features—such as high-frequency viral load trajectories, objective adherence measures, and inflammatory biomarkers—could provide signals that distinguish transient fluctuations from sustained immunological change (26). Alternative modelling formulations also warrant exploration: (1) predicting CD4^+^and CD8^+^counts separately before calculating the ratio, allowing tailored feature sets for each component; (2) using distributional regression (e.g., gamma) to better accommodate the ratio’s skewed nature; or (3) reframing ratio recovery as a time-to-event outcome (e.g., time to ratio >0.4) for greater clinical interpretability. Finally, uncertainty quantification techniques (e.g., conformal prediction) could flag low-confidence predictions for clinical review (27). While current ratio predictions are not yet reliable for individual-level decisions, they may still inform population-level monitoring and risk stratification. The insights gained from analysing these limitations directly guide the development of next-generation predictive tools for HIV immune monitoring.

To contextualize our findings, we compare our methodology and results with recent studies applying machine learning to HIV-related immunological outcomes. **Table 3** provided a comparative overview of our study alongside recent published work applying machine learning to HIV-related immunological prediction. While direct performance comparisons are complicated by differences in study populations, outcome definitions, and evaluation metrics, the table highlights the distinct features of our approach—including the exclusive use of baseline immunological values for cohort definition only (not as model inputs), the application of a heterogeneous stacking ensemble, and the multi-task prediction of three interrelated immunological outcomes. Li et al. developed Random Forest, SVM, and MLP models to predict immune function changes in 96 Chinese HIV patients over 9.9 years of ART, using routine clinical markers as inputs (22). Their models achieved significant correlations for CD4/CD8 ratio prediction (R up to 0.898), though direct performance metrics such as R^2^ or MAE were not reported. In contrast, our study leverages a substantially larger cohort (n=4,620) and a heterogeneous stacking ensemble, achieving an R^2^ of 0.786 for CD4^+^count and 0.636 for CD8^+^count on an independent test set—providing quantitative benchmarks that can inform future comparisons. Montesi et al. employed Random Forest regression to predict humoral responses to SARS-CoV-2 vaccination in 497 people living with HIV, achieving a cross-validated R^2^of 0.845 (23). While their study focused on vaccine immunogenicity rather than long-term immune reconstitution, it similarly underscores the value of non-linear algorithms in capturing complex immune dynamics. Our work extends this paradigm by demonstrating that stacking multiple algorithms can further improve predictive performance (66.4% and 128.6% R^2^ gains over a Transformer baseline for CD4^+^and CD8^+^counts, respectively), particularly under the stringent constraint of excluding baseline immunological markers. To our knowledge, no prior study has applied a heterogeneous stacking ensemble to predict long-term CD4^+^and CD8^+^trajectories in HIV while explicitly preventing data leakage by excluding baseline counts from model inputs. The systematic benchmarking against a state-of-the-art deep learning architecture under identical conditions further distinguishes our work. These comparisons position our framework as a robust and rigorously validated tool for clinical forecasting, with clear translational potential for personalized HIV care.

**Table 3:** Comparison of machine learning studies in HIV immunological prediction.

Study	Cohort/setting	Outcome(s)	Model(s)	Baseline CD4^+^/CD8^+^ as model input	Key performance (reported)
Li et al., 2022 (22)	Yunnan, China (n=96)	CD4^+^ count, CD8^+^ count, CD4/CD8 ratio	SVM, Random Forest, MLP	Yes (baseline values included)	R up to 0.898 for CD4/CD8 ratio; direct R^2^/MAE not reported
Montesi et al.,2024 (23)	Italy (n=497)	Anti-SARS-CoV-2 IgG (vaccine response)	Random Forest, Tree Regression, GLM	Yes (CD4^+^ count, CD4/CD8 ratio)	CV-R^2^ = 0.845 (Random Forest)
Maskew et al.,2022 (25)	South Africa (n=445,636)	Visit attendance, viral suppression	Logistic regression, Random Forest, AdaBoost	Not applicable (outcomes differ)	AUC 0.69–0.76
our study	Xi’an, China (n=4,620)	CD4^+^ count, CD8^+^ count, CD4/CD8 ratio	Stacking ensemble (XGBoost, LightGBM, Random Forest, Gradient Boosting + Ridge meta-learner)	No (excluded to prevent data leakage)	CD4^+^: R^2^ = 0.786 (MAE 74.8 cells/μL); CD8^+^: R^2^ = 0.636 (MAE 300.5 cells/μL); Ratio: R^2^ = 0.131 (MAE 0.137)

GLM, generalized linear model; MLP, multi-layer perceptron; SVM, support vector machine; CV, cross-validated.

Several limitations of this study warrant careful consideration. First, the model was developed and validated using data from a specific multicentre cohort within a defined treatment era (2016–2025). Its performance may vary when applied to populations with differing demographic profiles, transmission patterns, or regional ART guidelines, necessitating external validation across diverse clinical settings (21). Second, while the model demonstrated strong predictive accuracy in a retrospective, held-out test set, its ultimate clinical utility must be established through prospective validation in real-world practice. A prospective trial would assess its impact on clinical decision-making, patient outcomes, and healthcare efficiency. Third, although our analysis incorporated a broad set of demographic and clinical features, predictive performance could potentially be enhanced by integrating additional dynamic data streams. Future iterations could include medication adherence metrics (e.g., from pharmacy refills or electronic drug monitoring), viral genomic data (e.g., drug resistance mutations), comorbid disease status, and inflammatory biomarkers (e.g., hs-CRP, IL-6), which are known to influence immune recovery and clinical outcomes (5, 7). Fourth, the interpretability of the ensemble model presents a challenge. While it outperforms traditional linear models, its “black-box” nature complicates the direct explanation of individual predictions to clinicians. Future work should integrate *post-hoc* interpretability techniques (e.g., SHAP values, LIME) to provide clinicians with intuitive, case-specific reasoning, thereby fostering trust and facilitating integration into clinical workflows (28).

Several promising research directions emerge from this work. First, integrating additional data modalities—such as host genetic information, high-frequency viral load trajectories, and objective medication adherence patterns (e.g., from digital pillboxes or pharmacy refill records)—could further refine prediction accuracy and personalization. Second, developing robust model-agnostic interpretability tools is crucial to identify the most influential clinical features and temporal patterns driving predictions, thereby building clinician trust and facilitating adoption in routine care. Third, prospective validation studies in diverse clinical settings are essential to rigorously demonstrate the framework’s real-world utility and impact on patient management. Fourth, we acknowledge that our benchmark comparison was limited to a single deep learning baseline (Robust Transformer). Future work should expand the evaluation to include a broader range of comparator models commonly used in longitudinal HIV research, such as linear mixed-effects models, alternative machine learning approaches (e.g., bagging and boosting variants), and other recent deep learning architectures for clinical time-series forecasting. Such comparisons would further contextualize the performance of our ensemble framework relative to the state of the art. Finally, extending the predictive scope to other clinically relevant endpoints—such as the risk of specific opportunistic infections, incidence of non-AIDS comorbidities, or probability of virological failure—could significantly enhance the model’s comprehensive value in guiding personalized HIV care pathways.

Several factors should be considered when interpreting the generalizability of our findings. The study cohort was predominantly male (92.6%) and recruited exclusively from hospitals in Xi’an, China, with HIV transmission routes dominated by homosexual and heterosexual contact. These demographic and geographic characteristics may limit the applicability of our model to other HIV-positive populations with different epidemiological profiles—such as persons who inject drugs, key populations in sub-Saharan Africa, or patients with very low CD4 nadir (<50 cells/μL) who may exhibit distinct immune recovery dynamics. Immune reconstitution pathways can vary substantially across populations due to differences in baseline health status, comorbidities, access to care, and background immunogenetic factors. Therefore, while our framework demonstrates robust internal validity, external validation in diverse geographic and demographic settings is essential before considering clinical implementation. Future work should prioritize prospective multicentre studies that include underrepresented populations to assess the model’s performance and fairness across the full spectrum of people living with HIV.

The deployment of AI models in clinical practice necessitates rigorous attention to ethical and implementation considerations. In this study, all analyses were conducted using de-identified data with robust technical and administrative safeguards to ensure patient privacy and data security, adhering to established guidelines for secondary use of clinical data. It is imperative to emphasize that model predictions are designed to function as clinical decision support tools, augmenting rather than replacing clinician judgment. Successful integration into care workflows will therefore require targeted training programs to equip healthcare providers with the skills to critically interpret model outputs, understand their limitations (including uncertainty estimates), and apply them effectively within a holistic patient management context. Furthermore, ongoing monitoring for potential algorithmic bias and performance drift across different patient subgroups will be essential to ensure equitable and sustained clinical utility.

## Conclusion

In this study, we developed and validated a heterogeneous stacking ensemble learning framework for predicting longitudinal CD4^+^ T-cell recovery trajectories in patients living with HIV on ART. The model demonstrated robust and clinically relevant predictive performance across multiple immunological markers by strategically integrating the complementary strengths of diverse machine learning algorithms. This approach provides a scalable and accurate computational foundation for enabling personalized treatment monitoring and data-driven clinical decision support. While prospective validation in diverse clinical settings is essential to confirm its real-world utility, the proposed framework represents a significant step toward leveraging advanced artificial intelligence to enhance the precision and individualization of long-term HIV care, with the potential to improve clinical outcomes and optimize resource allocation. Future work should focus on external validation, model interpretability, and integration with evolving clinical data streams to realize its full translational potential.

## Data Availability

The raw data supporting the conclusions of this article will be made available by the authors, without undue reservation.
